# Cardiac sympathetic innervation in Parkinson’s disease versus multiple system atrophy

**DOI:** 10.1007/s10286-022-00853-6

**Published:** 2022-02-11

**Authors:** Christine Eckhardt, Florian Krismer, Eveline Donnemiller, Sabine Eschlböck, Alessandra Fanciulli, Cecilia Raccagni, Sylvia Bösch, Katherina Mair, Christoph Scherfler, Atbin Djamshidian, Christian Uprimny, Bernhard Metzler, Klaus Seppi, Werner Poewe, Stefan Kiechl, Irene Virgolini, Gregor K. Wenning

**Affiliations:** 1 Department of Neurology, Medical University of Innsbruck, Anichstraße 35, 6020 Innsbruck, Austria; 2 Department of Nuclear Medicine, Medical University of Innsbruck, Innsbruck, Austria; 3 Department of Neurology, San Maurizio Regional Hospital, Bolzano, Italy; 4 Department of Internal Medicine, Cardiology and Angiology, Medical University Innsbruck, Innsbruck, Austria; 5 Research Centre on Vascular Ageing and Stroke, VASCage, Innsbruck, Austria

**Keywords:** Parkinson’s disease, Multiple system atrophy, Parkinsonism, Cardiac sympathetic denervation, Autonomic function

## Abstract

**Purpose:**

The aims of this study were to evaluate the diagnostic accuracy of the dual imaging method combining cardiac iodine-^123^-metaiodobenzylguanidine single-photon emission computed tomography combined with low-dose chest computed tomography compared to routine cardiac scintigraphy, and assess regional differences in tracer distribution and the relationships between imaging and autonomic function in Parkinson’s disease and multiple system atrophy.

**Methods:**

A prospective study including 19 Parkinson’s disease and 12 multiple system atrophy patients was performed. Patients underwent clinical evaluation, iodine-^123^-metaiodobenzylguanidine single-photon emission computed tomography combined with chest computed tomography, planar scintigraphy, and cardiovascular autonomic function tests.

**Results:**

Co-registration of single-photon emission computed tomography and chest computed tomography resulted in three groups with distinct patterns of tracer uptake: homogeneous, non-homogeneously reduced and absent. There was a significant difference in group allocation among patients with multiple system atrophy and Parkinson’s disease (*p* = 0.001). Most multiple system atrophy patients showed homogeneous uptake, and the majority of Parkinson’s disease patients showed absent cardiac tracer uptake. We identified a pattern of heterogeneous cardiac tracer uptake in both diseases with reductions in the apex and the lateral myocardial wall. Sympathetic dysfunction reflected by a missing blood pressure overshoot during Valsalva manoeuvre correlated with cardiac tracer distribution in Parkinson’s disease patients (*p* < 0.001).

**Conclusions:**

The diagnostic accuracy of the dual imaging method and routine cardiac scintigraphy were similar. Anatomical tracer allocation provided by the dual imaging method of cardiac iodine-^123^-metaiodobenzylguanidine single-photon emission computed tomography and chest computed tomography identified a heterogeneous subgroup of Parkinson’s disease and multiple system atrophy patients with reduced cardiac tracer uptake in the apex and the lateral wall. Sympathetic dysfunction correlated with cardiac imaging in Parkinson’s disease patients.

## Introduction

The Parkinson variant of multiple system atrophy (MSA-P) and idiopathic Parkinson’s disease (IPD) are neurodegenerative disorders with distinct neuropathology and progression that share several clinical features hampering differential diagnosis, especially in the early stages of disease. While in IPD, degeneration of the cardiac postganglionic sympathetic nervous system is a characteristic finding, MSA-P patients show preganglionic abnormalities [1–9]. Cardiac imaging using iodine-123-metaiodobenzylguanidine (^123^I-MIBG), an inactive radiopharmaceutical sympathomimetic amine guanethidine, examines the sympathetic innervation of the heart and thereby enables the quantification of the postganglionic cardiac sympathetic innervation [10]. Accordingly, reduced cardiac ^123^I-MIBG uptake has been associated with IPD, even in the early stages, but is preserved in the majority of MSA cases [1–3, 9, 11–19]. A meta-analysis reported pooled sensitivity and specificity of 90% and 83%, respectively, for cardiac planar ^123^I-MIBG scintigraphy in differentiating parkinsonism associated with IPD from atypical parkinsonism [19]. Currently, ^123^I-MIBG scintigraphy is considered a supportive criterion within the diagnostic criteria of IPD [20]. However, up to 50% of de novo and early-stage IPD patients may show preserved cardiac sympathetic innervation, and reduced cardiac tracer uptake has been reported in one third of patients with MSA, consistent with a reduced number of postganglionic tyrosine hydroxylase immunoreactive axons and alpha-synuclein-positive inclusions in sympathetic ganglia of patients with MSA [17, 21–27]. Based on this knowledge gap, we sought to evaluate regional differences in cardiac tracer uptake in IPD and MSA-P. To this end, we applied co-registration of ^123^I-MIBG single-photon emission computed tomography and low-dose chest computed tomography (^123^I-MIBG-SPECT-CT), enabling us to assign functional information to anatomically defined cardiac territories. Secondly, we compared the diagnostic accuracy of ^123^I-MIBG-SPECT-CT to routine planar cardiac ^123^I-MIBG scintigraphy and determined the relationships between ^123^I-MIBG imaging and autonomic function.

## Materials and methods

### Study design and participants

We performed a cross-sectional study including 31 prospectively recruited patients with diagnosis of either probable MSA-P (*n* = 12) or probable IPD (*n* = 19) according to current consensus criteria [20, 28]. Medical records of all patients were counterchecked for diagnosis at the last visit at the outpatient clinics. All patients underwent a clinical interview and examination, echocardiography (ejection fraction > 54% for men, and > 52% for women), and planar ^123^I-MIBG scintigraphy followed by ^123^I-MIBG SPECT-CT. This study was approved by the ethics committee of the Medical University of Innsbruck (AN4687 311/4.25 [4307a]), and all participants signed the informed consent before inclusion in the study.

Exclusion criteria were a history of any other major neurological or psychiatric condition, a history of coronary heart disease, ischemic and non-ischemic cardiomyopathy, heart failure determined by echocardiography, cardiac denervation unrelated to parkinsonism (e.g. diabetic neuropathy or heart transplantation as determined by history or laboratory findings [i.e. HbA1c]), known or suspected pregnancy or breastfeeding, dependence on any drug known to interact with ^123^I-MIBG [29]. All patients were treated with levodopa alone or in combination with dopamine agonists.

### Clinical examination and rating scales

The clinical interview included collection of basic demographics, medical history and current medication. Disease severity and motor symptoms were evaluated using the Unified MSA Rating Scale (UMSARS) for patients with MSA-P and the Unified Parkinson’s Disease Rating Scale (MDS-UPDRS) for IPD and patients with MSA-P [30, 31].

### Cardiac ^123^I-MIBG imaging

The radiopharmaceutical agent AdreView™ (GE Healthcare) corresponding to ^123^I-MIBG is an agent used to functionally examine the sympathetic innervation of the heart. ^123^I-MIBG was applied according to the manufacturer’s guidelines. Four hours after application a planar overview scan of the thorax was recorded in each patient in a supine position (= planar ^123^I-MIBG scintigraphy), followed by a single SPECT-CT (= ^123^I-MIBG SPECT-CT). Photon attenuation correction was performed by CT. The gamma camera used for imaging was a Philips BrightView XCT with a cone beam CT instead of a spiral CT. The longer revolution time compared to a spiral CT means that respiratory movement will also lead to a blurring of the CT.

### ^123^I-MIBG SPECT-CT

To perform the synchronized image registration, the Philips BrightView XCT gamma camera system was used. It utilizes a flat-panel detector CT to achieve highest resolutions at low dosage. Low-dose CT imaging accounts for a radiation burden of 0.2 mSv according to manufacturer’s statements. The latter technology generates a resolution of 0.33 mm isotropic voxels. For image analysis, the obtained SPECT and low-dose CT image data were transferred to a Hermes Workstation (Hermes medical Solutions, Stockholm, Sweden) and displayed as 3-dimensional images using the multi-modality program Hybrid Viewer (Hermes medical Solutions, Stockholm, Sweden).

The first step was to manually place circular regions of interest (ROIs) comprising the entire left myocardial wall and the left ventricular chamber (ROI-H) and other circular ROIs comprising a representative part of the mediastinum (ROI-M) on the 2-dimensional coronal, transversal and sagittal sections for each patient. Inside these sectional ROIs the final volumes of interest of the heart (VOI-H) and the mediastinum (VOI-M) were generated by using the application data analysis VOI and the program “operation VOI threshold”. The former enables generating VOIs from the sectional ROIs and the latter enables the delineation of VOIs by digital thresholding based on voxel intensity within a defined range of tracer uptake intensity with a value set at 30%, which means all voxels above this value will be included in the VOIs. The value set represents counts per second per voxel.

Using these settings for each patient, different patterns of VOIs in the myocardial wall became visible and the patients were assigned to three distinct categories based on the tracer uptake profile:

Patients in group A had *homogeneous* ^123^I-MIBG uptake in the left ventricular wall and one VOI or two VOIs including the entire left ventricular wall were generated. Patients in group B had *non-homogeneously reduced* and partially absent ^123^I-MIBG uptake and more than two VOIs including portions of left ventricular wall were generated. Patients in group C had *absent* visible ^123^I-MIBG uptake in the left ventricular wall, which was discriminable from the background and no VOIs were generated.

In all patients, we determined the mediastinum as the reference VOI for the semi-quantitative evaluation. The mean counts per voxel of the final VOIs of the heart and the mediastinum were used to calculate the heart to mediastinum—VOI_Heart_/VOI_Mediastinum_ (VOI_H/M_) ratios.

The images of individuals of group B were inspected by one experienced nuclear medicine doctor (E.D.) and the tracer uptake was categorized into “normal”, reduced and absent within the territories the left ventricular wall.

### Planar ^123^I-MIBG scintigraphy

The regions of interest were defined on the heart myocardium and the mediastinum. The mean heart uptake/mean mediastinum uptake (H/M) were calculated.

### Cardiovascular autonomic function

Autonomic symptoms were evaluated using the COMPASS-31 and the SCOPA-AUT questionnaires [32, 33]. Sympathetic function was evaluated by assessment of the blood pressure (systolic and diastolic) and heart rate changes at 3-min tilt-table examination and active standing test, blood pressure (BP) overshoot during the Valsalva manoeuvre phase 4, and measurements of the total peripheral resistance (TPR) corresponding to peripheral sympathetic mediated vasoconstriction during tilt-table examination and active standing test [34, 35]. Orthostatic hypotension was diagnosed according to the consensus statement defining OH as a systolic blood pressure (BP) drop of at least 20 mmHg and/or a reduction in diastolic blood pressure of at least 10 mmHg at 3 min tilting or active standing [36]. Neurogenic orthostatic hypotension (nOH) was defined by a ratio < 0.5 of the increase in heart rate divided by the decrease in systolic blood pressure during tilt-table examination [37, 38]. Prior to the test, patients were asked to avoid coffee, tea or taurine-containing beverages and have their last meals 2 h before the scheduled test. The tilt-table battery was performed in a quiet setting, with mean 22 °C room temperature, following standardised protocols as described previously [39]. Briefly, heart rate (HR) and blood pressure (BP) were continuously monitored by non-invasive beat-to beat BP recording and impedance cardiography (Task Force Monitor, CNSystems 2007). After lying for 10 min in the supine position, patients were passively tilted up to 60° for 10 min. Oscillometric BP measurements were performed at the 10th minute of the supine phase and repeated 3 and 10 min after head-up tilting, and at 3 min during the active standing test. For analysis, we only considered the blood pressure, heart rate, and TPR changes at 3-min tilt-table examination and standing test. Valsalva manoeuvre included blowing into a mouthpiece for 15 s at an expiratory pressure of 40 mmHg, and three trials with 60-s intervals in between were performed.

### Statistical analyses

Statistical analyses were performed using IBM SPSS Statistics 25 software (SPSS, Inc., Chicago, IL, USA). All data are presented as mean ± standard deviation (SD) or as *n* (%). The Shapiro–Wilk test was applied to assess the distribution of data. For the two group comparisons between IPD and MSA-P, Mann Whitney *U* and *t* tests, and for the three group comparisons between the cardiac tracer uptake groups A–C, Kruskal Wallis test and ANOVA were conducted with post hoc Bonferroni test as appropriate. Frequencies and group differences in qualitative data were computed using cross-tabs and Chi-square test. Fisher’s exact test was applied for small sample sizes. Linear regression was performed to evaluate the relationship between heart to mediastinum ratios, diagnosis, disease duration, age, gender and a diagnosis of OH. Receiver operating curves were calculated for and compared between cardiac ^123^I-MIBG SPECT-CT and planar scintigraphy. Relationships between clinical and radiological data were performed using Pearson and Spearman correlations. Corrections for multiple comparisons were performed. The level of significance was set at *p* < 0.05.

## Results

### Demographic and clinical characteristics

Results of demographic and clinical characteristics are reported in Table 1.

**Table 1:** Demographic and clinical characteristics

*n* = 31	IPD	MSA-P	*P*
	*n* = 19	*n* = 12	
Age at examination (years)	65.37 (7.80)	63.33 (8.52)	*p* = 0.500°
Disease duration (months)	82.74 (29.63)	56.17 (26.09)	***p* = 0.017**°
Hoehn and Yahr stage (2/3/4)	11/8/0	0/9/3	***p* < 0.001**^Δ^
Gender (F:M)	5:14	11:1	***p* < 0.001**^**§**^
MDS-UPDRS total	72.61 (13.51)	96.00 (20.02)	***p* = 0.003**°
MDS-UPDRS I	11.39 (5.25)	17.14 (6.49)	***p* = 0.030**°
MDS-UPDRS II	15.56 (4.74)	21.59 (4.85)	***p* = 0.007**°
MDS-UPDRS III	40.39 (10.31)	52.86 (11.75)	***p* = 0.007**°
MDS-UPDRS IV	5 (1.5–9)	0 (0–8)	*p* = 0.298^Δ^
OH, *n* (%)	5/16 (31)	4/12 (33)	*p* = 0.612^**§**^
SCOPA-AUT gastrointestinal	3.5 (2.35)	8 (3.87)	***p* = 0.003** ^Δ^
SCOPA-AUT urinary	4.57 (4.55)	10 (6.23)	***p* = 0.018**^Δ^
SCOPA-AUT cardiovascular	1.07 (1.69)	2.73 (2.15)	***p* = 0.024**^Δ^
SCOPA-AUT total	13.79 (8.22)	21.47 (± 8.77)	***p* ≤ 0.001**°
COMPASS-31 urinary	1.51 (1.77)	3.95 (2.89)	***p* = 0.039**^Δ^
VOI_H/M_^123^I-MIBG SPECT-CT	1.1 (0.97–2.40)	4.23 (2.81–4.82)	***p* < 0.001**^Δ^
H/M planar scintigraphy	1.18 (0.19)	1.79 (0.34)	***p* < 0.001**°
Group A, *n* (%)	2/19 (11)	8/12 (67)	***p* = 0.001**^**§**^
Group B, *n* (%)	4/19 (21)	3/12 (25)	
Group C, *n* (%)	13/19 (68)	1/12 (8)	
Group B + group C, *n* (%)	17/19 (90)	4/12(33)	***p* = 0.002**^**§**^

Data for IPD and patients with MSA-P are reported as mean (SD), median (25–75% quartile), numbers and percentage within diagnosis. The two groups were compared using Chi-square (§), Mann Whitney *U* (Δ), and *t* tests (°)

*P* designates the group test *p*-value, *IPD* idiopathic Parkinson’s disease, *MSA-P* multiple system atrophy Parkinson-variant, *MDS-UPDRS* Movement Disorder Society Unified Parkinson’s Disease Rating Scale, *OH* orthostatic hypotension, *SCOPA-AUT* scales for outcomes in Parkinson’s disease—autonomic dysfunction, *COMPASS-31* Composite Autonomic Symptom Score-31, *VOI*_*H/M*_ heart to mediastinum ratio of the volume of interest of ^123^I-MIBG SPECT-CT, *H/M* heart to mediastinum ratio of the planar scintigraphy

While disease duration was significantly shorter in patients with MSA-P compared to IPD (*p* = 0.017), motor impairment was greater in patients with MSA-P, with higher MDS-UPDRS scores and a greater proportion of advanced H&Y stages (*p* < 0.001). The UMSARS scores of the MSA-P group were as follows: UMSARS total 46.67 ± 9.17, UMSARS I 20.42 ± 4.83, UMSARS II (motor) 23.33 ± 4.03, UMSARS global 2: *n* = 3, 3: *n* = 3, 4: *n* = 5 (median 3 [CI 2.51–3.66]). In the MSA-P group the female gender was predominant. There was no difference in the frequency of orthostatic hypotension in patients with IPD or MSA-P (*p* > 0.05). Statistically significant differences were observed in the total SCOPA-AUT scores between patients with IPD and MSA-P (*p* < 0.001). The latter had higher scores on the bowel function, urinary and cardiovascular domains (*p* < 0.05, Table 1). The score in the urinary domain of COMPASS-31 was also higher in the MSA-P compared to the IPD patients (*p* = 0.039). The other domains of COMPASS-31 were comparable between IPD and patients with MSA-P.

### ^123^I-MIBG SPECT-CT

**Fig. 1:**
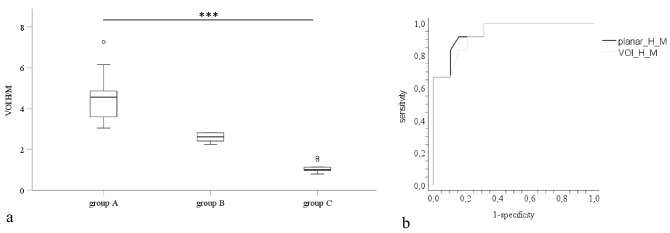
VOI_H/M_ of ^123^I-MIBG SPECT-CT of groups A, B and C are illustrated as boxplots (**a**). The ROC curves of VOI_H/M_ of ^123^I-MIBG SPECT-CT (grey) and planar scintigraphy (black) are shown on the right side (b). **a** The three groups were compared using Kruskal–Wallis and appropriate post hoc test. VOI_H/M_ differed significantly between groups A, B and C (for all group comparisons *p* < 0.001). **b** The area under the curve for the VOI_H/M_ of ^123^I-MIBG SPECT-CT was 0.934; the AUC for the planar scintigraphy was 0.945 (*p* = 0.350). VOI_H/M_ = heart to mediastinum ratio of the volume of interest. Group A = homogeneous, group B = non-homogeneously reduced, and group C = absent cardiac ^123^I-MIBG tracer uptake

Results of the VOI_H/M_ of ^123^I-MIBG SPECT-CT and the ROC curves of VOI_H/M_ of ^123^I-MIBG SPECT-CT and planar scintigraphy are plotted in Fig. 1.

There were three categories of cardiac ^123^I-MIBG tracer uptake identified by SPECT-CT: Group A consisted of 10 patients with homogeneous tracer uptake and was dominated by eight patients with MSA-P. Group B consisted of seven patients showing non-homogeneously reduced tracer uptake including three patients with MSA-P and four IPD patients. All patients of group C with absent cardiac tracer uptake were IPD patients, except one patient with MSA-P (Table 1). Comparison of the diagnostic accuracy of the dual imaging method of ^123^I-MIBG SPECT-CT to planar scintigraphy did not show any significant difference (Fig. 1b).

VOI_H/M_ of ^123^I-MIBG SPECT-CT and H/M of planar scintigraphy were significantly higher in MSA-P than in IPD (*p* < 0.001), and there was a significant association of the tracer uptake group and diagnosis (*p* < 0.01; Table 1). Overall, the planar scintigraphy-derived H/M ratio strongly correlated with the SPECT-CT-derived averaged VOI_H/M_-ratio (*r* = 0.942, *p* < 0.001 *n* = 31). A total of 89% of IPD and 33% of patients with MSA-P revealed a pathological MIBG scan (absent or reduced) (*p* = 0.001; Table 1). Absent or reduced tracer uptake resulted in an odds ratio (OR) of 17 for the diagnosis of IPD (sensitivity 89% and specificity 67%, accuracy 81%, the negative likelihood ratio for a diagnosis of IPD in the context of homogeneous cardiac MIBG uptake was 0.16). Missing cardiac tracer uptake was significantly associated with a diagnosis of IPD (*p* = 0.0003); the corresponding OR was 24 (sensitivity 93% and specificity 65%, accuracy 77%).

Myocardial tracer uptake was not correlated with age, gender, disease duration or disease severity (*p* > 0.05). Linear regression was carried out to investigate the relationship between VOI_H/M_ and diagnosis, age at examination, disease severity (H&Y), gender and a diagnosis of OH. A strong association was identified between VOI_H/M_ and diagnosis (*n* = 28; only diagnosis was a significant predictor: regression coefficient 2.63; beta = 0.77; *p* = 0.005).

### Patterns of regional differences ^123^I-MIBG uptake in patients with non-homogeneously reduced tracer uptake.

**Fig. 2:**
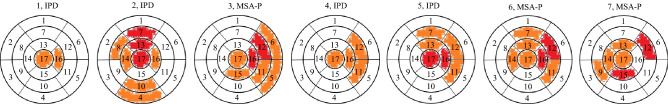
The patterns of cardiac 123I-MIBG tracer uptake of patients 1–7 of group B are illustrated. The numbers in the images reflect the 17 cardiac territories as follows: 1: basal anterior, 2: basal anteroseptal, 3: basal inferoseptal, 4: basal inferior, 5: basal inferolateral, 6: basal anterolateral, 7: mid anterior, 8: mid anteroseptal, 9: mid inferoseptal, 10: mid inferior, 11: mid inferolateral, 12: mid anterolateral, 13: apical anterior, 14: apical septal, 15: apical inferior, 16: apical lateral, 17; apical. red = completely, orange = incompletely reduced tracer uptake. Plain spaces indicate normal tracer uptake

The patterns of regional tracer distribution in the patients of group B are illustrated in Fig. 2.

Four IPD and three patients with MSA-P were assigned to group B (three men and one female of the IPD and two female and one man in the MSA-P group). Reduced cardiac tracer uptake was most common in the apex (segment 17) and the lateral wall (segments 5, 6, 11, 12, 16) detected in all seven patients of group B, followed by the anterior wall (segments 1, 7, 13) detected in 4/7 patients (57%), the septal wall (segments 2, 3, 8, 9, 14) observed in 3/7 patients (43%), and the inferior wall (segments 4, 10 and 15) found in 3/7 patients (43%).

### Cardiovascular autonomic function and cardiac ^123^I-MIBG uptake

Cardiovascular autonomic function parameters are summarized in Table 2.

**Table 2:** Cardiovascular autonomic function parameters

	MIBG tracer uptake			*P*
	Group A	Group B	Group C	
OH, *n* (%)	3/9 (33) 3 MSA-P	1/9 (11) 1 IPD	5/9 (56) 1 MSA-P, 4	*p* = 0.758§x
Without OH, *n* (%)	7/19 (37) 2 IPD, 5 MSA-P	6/19 (32) 3 IPD, 3 MSA-P	IPD 6/19 (32) 6 IPD	
nOH, *n* (%)	3/6 (50) 3 MSA-P	1/6 (17) 1 IPD	2/6 (33) 1 MSA-P, 1 IPD	*p* = 0.330§x
Valsalva				
Missing BP overshoot phase 4, *n* (%)	5/18 (28)	3/18 (17)	10/18 (56)	*p* = 0.448§x
Tilt-table examination				
Δ systolic BP 3 min [mmHg]	−5.60 (10.11)	−11.71 (24.19)	−8.09 (19.44)	*p* = 1#x
Δ diastolic BP 3 min [mmHg]	3.00 (8.79)	−4.29 (18.29)	0.00 (14.78)	*p* = 1#x
Δ HR 3 min [beats/min]	7.80 (5.96)	6.86 (7.47)	11.09 (6.86)	*p* = 1#x
Δ TPR 3 min	455 (213)	220 (634)	112 (379)	*p* = 1#x
Active standing test				
Δ systolic BP 3 min [mmHg]	3.50 (21.90)	6.33 (18.08)	0.45 (24.61)	*p* = 1#x
Δ diastolic BP 3 min [mmHg]	8 (−1.75–22.25)	12.5 (6.25–24.5)	15 (−10–21)	*p* = 1^x
Δ HR 3 min [beats/min]	12.70 (7.60)	15.00 (4.82)	17.00 (6.37)	*p* = 1#x
Δ TPR 3 min	442 (329)	455 (393)	13 (353)	*p* = 0.144x

Data for groups A, B and C (group A = homogeneous; group B = non-homogeneously reduced; group C = absent) are reported as mean (SD), median (25–75% quartile), numbers and percentages. The three groups were compared with Chi-square (§), ANOVA (#), or Kruskal Wallis (^) tests with appropriate post hoc tests and correction for multiple comparisons (x)

*P* designates the overall group test *p*-value, *OH* orthostatic hypotension, *nOH* neurogenic OH, *BP* blood pressure, *HR* heart rate, *TPR* total peripheral resistance

Orthostatic hypotension was diagnosed in 9/28 (32%) patients (three patients were excluded from analyses): 5/16 (31%) of IPD and 4/12 (33%) of patients with MSA-P (*p* = 0.612, Table 1). The VOI_H_/VOI_M_ were comparable in patients with and without a diagnosis of OH (*p* = 0.735). Neurogenic OH (nOH) was diagnosed in six cases with OH (2/5 (40%) IPD and 4/4 (100%) MSA-P cases with OH; *p* = 0.119).

Orthostatic hypotension and nOH were not associated with the cardiac tracer uptake groups (*p* > 0.05; Table 2), and no significant correlations of HR, BP or TPR during tilt-table examination or standing test with VOI_H/M_ of ^123^I-MIBG SPECT-CT could be identified. More than 80% of IPD patients without OH (9/11 IPD patients) showed pathological cardiac tracer uptake: 6/11 (55%) with absent and 3/11 (27%) with reduced levels. In MSA-P 5/8 (62.5%) showed normal and 3/8 (37.5%) had reduced tracer uptake. Among the patients without a diagnosis of OH, there was a significant association of a diagnosis and tracer uptake category (*p* = 0.030; Table 2).

Impaired sympathetic function reflected by a missing BP overshoot during Valsalva phase 4 was observed in 10/14 (71%) IPD and 8/10 (80%) patients with MSA-P (*p* = 0.506). Considering all patients, no significant correlation of the missing blood pressure overshoot during Valsalva phase 4 and tracer uptake group could be determined. However, looking into the subsets, there was a highly significant correlation of tracer uptake group and a missing BP overshoot during Valsalva phase 4 in the IPD (*r* = 0.875 *p* < 0.001) but not in the patients with MSA-P (*p* = 1). This result was strengthened by a significant correlation of a missing BP overshoot during Valsalva phase 4 with the VOI_H/M_ in the IPD group (*r* = −0.746 *p* = 0.036) but not in the patients with MSA-P (*p* = 1).

## Discussion

This is the first study investigating (1) differences in regional tracer distribution using cardiac ^123^I-MIBG SPECT-CT, (2) its diagnostic accuracy compared to routine ^123^I-MIBG scintigraphy, and (3) its relationship with sympathetic function tests in IPD and patients with MSA-P.

In contrast to previous studies investigating cardiac sympathetic innervation in parkinsonism by planar ^123^I-MIBG scintigraphy, the dual imaging method combining ^123^I-MIBG SPECT and low-dose chest CT offers the possibility to map regional differences in cardiac tracer uptake indicative of sympathetic innervation [1–3, 9, 11–19]. Thereby, we established three distinct groups of cardiac ^123^I-MIBG tracer distribution with homogeneous (group A corresponding to normal cardiac tracer uptake), non-homogeneously reduced (group B, incomplete cardiac tracer uptake), and absent (group C) tracer uptake. The IPD patients dominated group C (68% of IPD patients), and the majority of patients with MSA-P belonged to group A (67% of patients with MSA-P). Pathological tracer uptake (groups B and C) was highly associated with a diagnosis of IPD, revealing an OR of 17 and corresponding to sensitivity of 89% and specificity of 67% (negative likelihood ratio 0.16), which is concordant with previous reports [1, 2, 12–15, 19]. Twenty-one percent of IPD patients and one third of patients with MSA-P were assigned to group B with non-homogeneously reduced cardiac tracer uptake. In group B, the apex and the lateral wall of the myocardium were most commonly affected, irrespective of the underlying disease pathology. A similar pattern was identified by fluorine-18-fluorodopamine and 11C-hydroxyephedrine positron emission studies in IPD patients [40, 41]. In contrast, Lebasnier et al. showed that the inferior wall of the myocardium was most affected by reduced tracer uptake in patients with Parkinson’s disease and dementia with Lewy bodies, followed by the apical, lateral, septal and anterior walls, and they speculated that regional cardiac sympathetic denervation is caused by Lewy body deposition [42]. However, other mechanisms may be involved, including physiological variation and liver and lung interference. Several reports have pointed out that sympathetic innervation in healthy individuals is not uniform, but results are conflicting [43–57]. Most commonly, reductions in cardiac MIBG tracer uptake have been reported in the apex and the inferior wall of healthy individuals with associations to age and gender [45, 46]. No linkage between the VOI_H/M_ and age or gender could be identified in our study. Histological quantification methods of post-mortem human tissue assessing cardiac sympathetic innervation are limited. In a study by Kawano and co-workers, the anterior wall of the left ventricle showed significantly higher density of adrenergic innervation compared to the inferior wall in healthy individuals [56], supporting the differences found by imaging studies. Nevertheless, it remains unclear whether these reductions accounting for approximately 20% can really explain the imaging differences, knowing that regional denervation needs to be severe before it becomes apparent in MIBG imaging [55]. Notably, the reductions in MIBG tracer uptake in the lateral wall of the left ventricle detected in the present study differ from what has been reported in healthy individuals and patients with IPD or dementia with Lewy bodies. Future studies assessing the temporal sequence of ^123^I-MIBG SPECT-CT imaging combined with post-mortem studies could possibly clarify the evolution of sympathetic denervation. Moreover, histochemical analyses could resolve metabolic and storage deficiencies potentially contributing to the heterogeneity of tracer metabolism. Finally, a larger number of patients with regional differences in cardiac MIBG imaging may offer the possibility to more reliably distinguish PD patients from patients with MSA-P by their tracer uptake profile.

The proportion of patients with MSA-P (33%) with pathological tracer uptake has been reported in previous studies [24, 25], and there is literature indicating histopathological postganglionic abnormalities in patients with MSA [17, 27].

As expected, the VOI_H/M_^123^I-MIBG SPECT-CT and H/M of planar scintigraphy were higher in the patients with MSA-P than in the IPD patients. No correlations of VOI_H/M_^123^I-MIBG SPECT-CT and age, disease duration, disease severity, or blood pressure changes during cardiovascular autonomic function tests were determined, which has been shown before but contrasts with other reports [24, 59]. Despite the beneficial aspect of regional tracer mapping, the diagnostic accuracies of VOI_H/M_^123^I-MIBG SPECT-CT and H/M of planar scintigraphy were comparable in this study.

It has been demonstrated that OH in IPD is associated with neuroimaging evidence of cardiac sympathetic denervation in IPD patients [58]. We could not reproduce this association between OH and tracer uptake category, which may be because of the small number of IPD patients with a diagnosis of OH in the current study (*n* = 5). However, we were able to confirm that a diagnosis of IPD is associated with pathological tracer uptake in the absence of OH (80%). Further, we show that cardiac sympathetic innervation assessed by the VOI_H/M_ of ^123^I-MIBG SPECT-CT correlates with sympathetic function tests [59]: the missing blood pressure overshoot during the Valsalva manoeuvre phase 4 was associated with absent cardiac tracer uptake and correlated with the VOI_H/M_^123^I-MIBG SPECT-CT in the IPD patients. A missing BP overshoot during phase 4 of the Valsalva manoeuvre indicates impaired sympathetic control over peripheral blood vessels. Given that the main site of lesions in the autonomic nervous system in people with IPD is at the level of post-ganglionic noradrenergic fibres, the observed association between absent cardiac ^123^I-MIBG tracer uptake and missing blood pressure overshoot at Valsalva manoeuvre phase 4 suggests that sympathetic impairment proceeds at a similar pace at the cardiac and vascular levels in people with IPD. This could not be shown for the MSA-P group, which can be explained by predominantly preganglionic sympathetic degeneration in people with MSA [60].

In the light of controversial reports regarding the relationships between a diagnosis of OH, blood pressure and heart rate changes during autonomic function test and cardiac ^123^I-MIBG imaging, we did not find any significant correlations in this study.

The severity of autonomic features as measured by the SCOPA-AUT and COMPASS-31 questionnaires did not correlate with clinical characteristics or cardiac^123^I-MIBG imaging as reported previously [23, 61, 62].

Very recently, a model of “brain first” versus “body first” Parkinson’s disease was introduced by abnormal cardiac ^123^I-MIBG scintigraphy present before loss of putaminal dopamine storage capacity in IPD patients with REM sleep behaviour disorder (RBD) and idiopathic RBD in contrast to IPD patients without RBD, revealing deficits in dopamine storage prior to cardiac sympathetic denervation [63]. In the current study population, all except two IPD patients showed pathological cardiac tracer uptake representing the proposed final common path in almost all IPD patients [21, 22]. The two IPD cases with unremarkable cardiac MIBG imaging (age: 69 and 53 years, disease duration: 53 and 84 months; H&Y stage: 3 and 2) might be assigned to the “brain first” model of Horsager and coworkers [63].

Confounding factors such as cardiac insufficiency, cardiomyopathy, cardiac denervation unrelated to parkinsonism, or medication known to possibly interfere with ^123^I-MIBG imaging were excluded in this study [29].

We acknowledge several limitations: The cross-sectional design of the current study lacks information about the evolution of cardiac tracer uptake which would be of high interest in the patients with non-homogeneously reduced cardiac tracer distribution. The sample size was modest, especially in group B, and the majority of patients were female. There was no post-mortem confirmation of clinical diagnosis of IPD or MSA-P. Cardiac tracer uptake categories including homogeneous, non-homogeneously reduced, and absent were established based on visual inspection of the SPECT-CT images. Advanced statistical algorithms including supervised and unsupervised machine learning approaches may reveal additional patterns that were not recognized during visual reading in our study and should be further investigated in future studies.

In conclusion, this is the first study offering a map of cardiac ^123^I-MIBG tracer allocation provided by the dual imaging method of ^123^I-MIBG SPECT-CT in IPD and patients with MSA-P. In both diseases, the apex and the lateral wall of the myocardium are most affected by reduced tracer uptake in patients with non-homogeneous reductions in ^123^I-MIBG uptake. In IPD, cardiovascular sympathetic function indicates postganglionic imaging but is not associated with OH. Finally, the diagnostic accuracy of ^123^I-MIBG SPECT-CT is comparable to routine ^123^I-MIBG scintigraphy. Future longitudinal studies assessing cardiac ^123^I-MIBG tracer abnormalities in IPD and MSA patients with and without neurogenic OH and advanced statistical analyses including automated pattern recognition would be highly desirable, as such studies might help to better characterize the topographical evolution of cardiac noradrenergic denervation in neurodegenerative disorders.

## Data Availability

The datasets generated during and analysed during the current study are not publicly available but are available from the corresponding author on reasonable request.
